# Activation of group II metabotropic glutamate receptors blocks zinc release from hippocampal mossy fibers

**DOI:** 10.1186/0717-6287-47-73

**Published:** 2014-12-18

**Authors:** Carlos M Matias, Jose C Dionísio, Peter Saggau, Maria Emilia Quinta-Ferreira

**Affiliations:** Center for Neurosciences of Coimbra, University of Coimbra, 3004-516 Coimbra, Portugal; Department Animal Biology, FCUL, University Lisbon, Campo Grande, Lisbon, Portugal; Allen Institute for Brain Science, 551 N 34th Street, Seattle, USA; Department of Physics, University of Coimbra, 3004-516 Coimbra, Portugal; Department of Physics, School of Science and Technology, University of Trás-os-montes and Alto Douro (UTAD), Quinta dos Prados, 5000-911 Vila Real, Portugal

**Keywords:** Zinc, Neurotransmission, Glutamatergic, Co-release, Metabotropic fluorescence, Inhibition

## Abstract

**Background:**

The hippocampal CA3 area contains large amounts of vesicular zinc in the mossy fiber terminals which is released during synaptic activity, depending on presynaptic calcium. Another characteristic of these synapses is the presynaptic localization of high concentrations of group II metabotropic glutamate receptors, specifically activated by DCG-IV. Previous work has shown that DCG-IV affects only mossy fiber-evoked responses but not the signals from associational-commissural afferents, blocking mossy fiber synaptic transmission. Since zinc is released from mossy fibers even for single stimuli and it is generally assumed to be co-released with glutamate, the aim of the work was to investigate the effect of DCG-IV on mossy fiber zinc signals.

**Results:**

Studies were performed using the membrane-permeant fluorescent zinc probe TSQ, and indicate that DCG-IV almost completely abolishes mossy fiber zinc changes as it does with synaptic transmission.

**Conclusions:**

Zinc signaling is regulated by the activation of type II metabotropic receptors, as it has been previously shown for glutamate, further supporting the corelease of glutamate and zinc from mossy fibers.

## Background

Zinc is one of the most influent transition metals present in the brain and has an important role in various neuronal processes, such as protein activation and neurotransmission [1, 2]. Free or weakly-bound zinc is sequestered in the synaptic vesicles of zinc-containing neurons, constituting most of the histochemically reactive zinc in the brain [3]. Some glutamatergic neurons, in particular the granule cells of the hippocampus contain large amounts of zinc, which is present in their synaptic terminals, the mossy fibers [3, 4]. Cell depolarization and subsequent calcium influx evoke zinc release from presynaptic vesicles [5] that interacts with multiple receptors and channels, either inhibiting or enhancing their responses [6–10]. It is generally assumed that zinc is co-released with glutamate, although no direct evidence exists for that assumption [4, 11]. Zinc is also released from mossy fibers following low levels of stimulation, which was measured with permeant fluorescent zinc indicators, such as TFLZn [12] and N-(6-methoxy-8-quinolyl)-para-toluenesulfonamide (TSQ) [13, 14] or the impermeant zinc dye FluoZin-3 [15]. The nature of TSQ zinc signals was tested using the very high-affinity and specific permeant zinc chelator,N,N,N,N'tetrakis(2-pyridylmethyl)ethylenediamine (TPEN), as well as the impermeant zinc chelator Ca-EDTA [16]. It was observed that these signals are abolished by TPEN but remain in the presence of Ca-EDTA.

However, it should be noted that stimulation of the mossy fiber pathway may also evoke electrical activity in a zinc-poor synaptic system, the associational/commissural fibers, which are also present in that region of the hippocampus [4, 17, 18]. Metabotropic glutamate (mGlu) receptors are involved in various synaptic functions in the central nervous system [19, 20]. The mossy fibers have very specific characteristics, including the presence of group II mGlu receptors, which inhibit glutamate release and, in consequence, synaptic transmission [21]. It was observed that application of a potent group II-selective mGlu receptor agonist (2S,1'R,2'R,3'R)-2-(2,3-dicarboxycyclopropyl)glycine(DCG-IV) reversibly and differentially reduced the field potentials and presynaptic calcium signals [17, 18, 22] in hippocampal slices. In order to observe the relationship between the effect of the activation of group II mGlu receptors and zinc transients obtained from mossy fiber stimulation, we have investigated the action of DCG-IV on presynaptic zinc signals, using the fluorescent zinc indicator TSQ. It is shown that the application of DCG-IV reversibly blocks zinc signals evoked by single stimuli, while the synaptic activity was also significantly reduced. These results indicate a relationship between glutamate and zinc release in mossy fibers, reinforcing the idea that zinc is co-released with glutamate.

Optical traces represent fractional changes in fluorescence (ΔF/F), where ΔF represents changes in fluorescence after stimulation and F the resting value of fluorescence, corrected for autofluorescence. All stimuli were given at baseline stimulus strength, which is the average of the first ten pulses delivered at the beginning of the experiment. Data are expressed as mean ± S.E.M. Statistical significance was evaluated using the Mann-Whitney *U* test (p < 0.05). Drugs used were: TSQ (Molecular Probes Europe BV, Leiden, NL) CNQX, D-APV and DCG-IV (Tocris Cookson, Bristol, UK).

## Results

**Figure 1:**
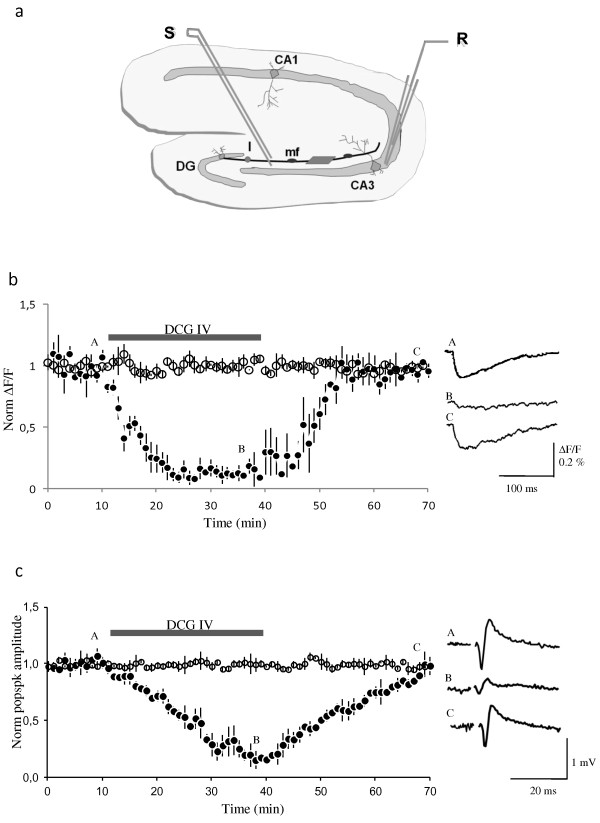
**Diagram of the hippocampal slice and presynaptic zinc signals and field potentials obtained in the presence of DCG-IV. a**. Hippocampal slice scheme representing different hippocampal areas, CA1, CA3 and dentate gyrus (DG), and the mossy fiber tract (mf). It also shows the location of the injection site (I) of the calcium indicator Fura-2, the stimulation electrode (S) and the recording electrode (R). The dark squared area represents the region from where the optical signals were detected. **b**. Presynaptic zinc signals evoked by single stimuli are significantly and reversibly reduced by the application of DCG-IV. Normalized amplitude of zinc signals from experiments with (closed circles) and without (open circles) the mGlu receptor agonist DCG-IV (left side), and sample zinc transients (*n* = 5), (right side). **c**. Normalized amplitude of the population spikes (left) and sample field potentials (right) from the same experiments. The sample traces A–C were recorded at the times indicated by the letters in the graphs. The bars represent the period of application of the solutions containing DCG-IV. The slices were stimulated at the control frequency (16 mHz). The points in the graphs represent the mean value ± S.E.M. All stimuli were delivered at baseline stimulus strength.

Figure 1 shows the results of experiments designed to determine the effect of the group II mGlu receptors agonist DCG-IV, on the zinc signals and on the corresponding field potentials. The upper part (Figure 1a) is a schematic representation of a hippocampal brain slice, with the main areas of the hippocampus, the electrode arrangement, and the region from where the optical signals were detected. The middle part (Figure 1b) shows the normalized pooled data of the zinc signals and also sample zinc transients. As can be seen, the zinc changes were almost completely and reversibly abolished by DCG-IV. In the presence of this drug, the amplitude of the zinc signals at 25-30 min after its application, decreased to 13 ± 8% (mean ± S.E.M., n = 5; p < 0.05) of baseline. The right side of this panel shows representative zinc traces measured before, during the application and after washout of DCG-IV. The lower part (Figure 1c) presents the effect of DCG-IV on the corresponding population spikes and also sample field potentials. In these experiments, the amplitude of the population spikes of the somatic field potentials was markedly reduced to 19 ± 5% (n = 7, p < 0.05) of baseline values, indicating a correspondence between the zinc transients and field potentials.

**Figure 2:**
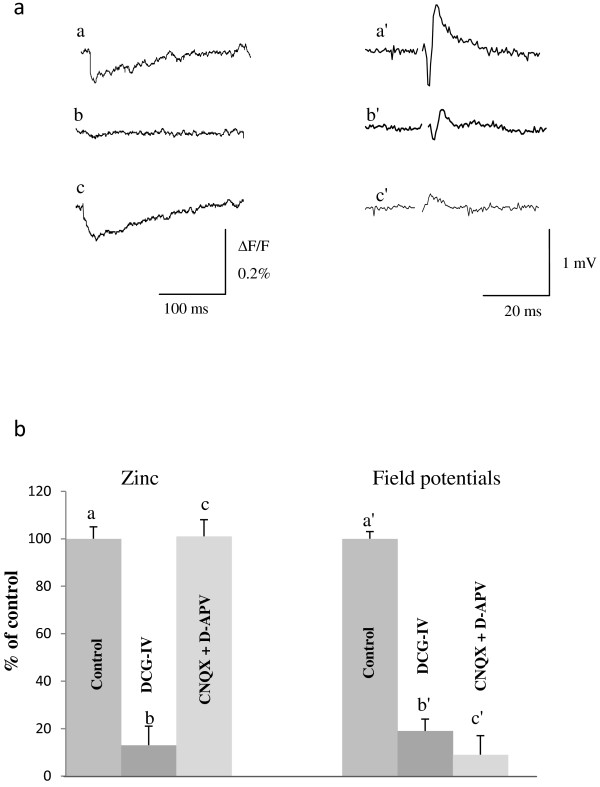
**Effect of DCG-IV and CNQX + D-APV on zinc and field potential signals. a**. sample zinc signals (left side) and field potentials (right side) evoked by single stimuli (n = 5), **b**. Bar graph representing the averaged amplitude of five records for each type of signals, obtained in ACSF (control), DCG-IV (1 μM) or CNQX (10 μM) + D-APV (50 μM). The bars represent the mean value ± S.E.M. All stimuli were given at baseline stimulus strength.

For a comparison of the effect of DCG-IV on zinc and field potentials, data representing the normalized amplitude of these signals, in the absence and presence of DCG-IV, is summarized on the bar graph of Figure 2. This figure includes also the effect of CNQX (10 μM) and D-APV (50 μM), antagonists of AMPA and NMDA receptors, respectively, on the same signals, in order to verify their pre- or postsynaptic nature.

The zinc transients were maintained in the presence of CNQX (10 μM) and D-APV (50 μM), which block AMPA and NMDA receptors, respectively, abolishing, as shown, synaptic transmission (Figure 2). In the presence of both drugs the normalized values of the zinc and field potentials were 98 ± 7% (n = 5), 101 ± 7% (n = 5) and 9 ± 8% (n = 7), respectively, of the control values obtained in ACSF. These observations indicate that both zinc and calcium transients are presynaptic.

## Discussion

In this study, we investigated the presynaptic inhibitory action at the hippocampal mossy fiber synapses, by group II mGlu receptors. We have observed that application of the group II mGlu receptors agonist DCG-IV has a similar effect on the presynaptic zinc signals and field potential responses induced by single stimuli, in agreement with the results obtained measuring extracellular zinc changes [15]. The degrees of inhibition of both the electrical and the calcium signals, being the first ones more affected by DCG-IV, are in agreement with those reported in previous works [17, 18, 23]. The calcium results, obtained using the fluorescent indicator Fura-2 [24], indicate that the amplitude of the calcium signals was reduced to 62 ± 8% of the control values (n = 5, p < 0.05), in the presence of DCG-IV. This inhibition is approximately three to four times smaller than that observed on both the zinc and field potential signals in this study, in accordance with an about fourth-power relationship between presynaptic calcium transient and vesicular transmitter release [25].

The suppression of glutamate release evoked by the activation of group II mGlu receptors in mossy fiber terminals, maybe, at least in part, due to the inhibition of forskolin-stimulated cyclic adenosine mono phosphate (cAMP) formation [26, 27]. It was observed that an increase in cAMP concentration within mossy fiber terminals leads to persistent enhancement of transmitter release [28, 29], suggesting that a decrease in cAMP concentration is likely to be involved in this DCG-IV induced inhibition of transmitter release process. Another possible contribution for the observed inhibition is the reduction of action potential-induced calcium influx into presynaptic terminals by DCG-IV, followed by the suppression of transmitter release. Previous observations indicate that mGlu receptor 2 activation can inhibit zinc relase [30] and N-type voltage-gated calcium channels [31, 32], which mediate glutamate release. Down-regulation of these channels, which are involved in synaptic transmission at the mossy fiber-CA3 synapses in the hippocampus [33], may result in the suppression of calcium-dependent glutamate release.

Since mossy fiber zinc and glutamate are assumed to be co-released, and both processes are calcium-dependent, the mentioned DCG-IV-evoked cAMP and N-type calcium channel processes are also expected to affect zinc release.

## Conclusions

The results show that the mGlu receptor agonist DCG-IV causes a large inhibition of both zinc release and synaptic transmission but a small reduction of the calcium transients. Thus, as observed for glutamate, synaptic zinc dynamics depends on the activation of presynaptic group II mGlu receptors.

## Methods

The experiments were performed on 4-6 weeks old Wistar rats. The animals were anesthetized with ethyl ether and quickly decapitated. Transverse brain slices (400 μm thick) were obtained from the hippocampus. They were then transferred to the experimental chamber where they were continuously perfused (1.5–2 ml/min), with oxygenated artificial cerebrospinal fluid (ACSF) at 30–32°C. The ACSF solution contained (in mM): NaCl 124; KCl 3.5; NaHCO_3_ 24.0; NaH_2_PO_4_ 1.25; MgCl_2_ 2.0; CaCl_2_ 2.0 and glucose 10.0. The stimulation was delivered by means of stainless steel bipolar electrodes placed on the mossy fibers of the dentate granule cells (DG), which form synapses with the proximal dendrites of CA3 neurons (Figure 1). Single current pulses (200-500 μA; 100 ms), equal to 40% of the saturation value, were applied every minute (at 16 mHz). These evoked field potentials that were recorded extracellularly at the pyramidal cell layer, using glass microelectrodes (1-10 MΩ) containing a 2 M NaCl solution. The zinc studies were performed in brain slices pre-loaded (60-90 min, 35-39°C) with the membrane-permeant fluorescent zinc indicator TSQ (30 μM), as previously described [14]. TSQ is characterized by a very low fluorescence in the free form, but a large intensity of fluorescence, without changes in wavelength, following zinc complexation. Thus, zinc release will lead to a decrease in the fluorescence of the TSQ/Zn complex, since the free zinc concentration in the synaptic cleft is about four orders of magnitude lower than inside the vesicles. Presynaptic calcium signals were detected using the permeant form of the fluorescent calcium indicator Fura-2, which was pressure-injected in the mossy fiber tract as reported before [23]. The optical signals were obtained in the region represented by the rectangle shown in Figure 1, using a silicon photodiode and an optical setup for transfluorescence measurements. Light was focused onto and collected from the brain slice, by means of two identical objective lenses (40x N.A. 0.75, Zeiss). For the zinc measurements, in which a tungsten/halogen lamp (12, 100 W) was used, the excitation (400 nm) and emission wavelengths were selected by means of a linear variable interference filter (400-700 nm; Schott), with slit widths of 0.6 mm, and a 500 nm long-band pass filter, respectively. The signal from the photodiode after passing through an I/V converter with a 1 GΩ feedback resistance, was applied into an AC-coupled amplifier with a low (1 Hz) cut-off frequency. The agonist of group II mGlu receptors DCG-IV (1 μM) and 6-cyano-7-nitroquinoxaline-2,3-dione (CNQX, 10 μM) and 2*R*-amino-5-phosphonopentanoate (D-APV, 50 μM), which block AMPA and NMDA receptor channels, were added to the perfusion medium, the latter ones at the end of the experiments. During the period (30 min) of application of these drugs solutions, which were recirculated (100 ml), control frequency stimulation (16 mHz) was delivered to the mossy fibers.

All experiments were carried out in accordance with the European Communities Council Directive. All efforts were made to minimize animal suffering and to use only the number of animals necessary to produce reliable scientific data.
